# Progress of elemental anomalies of hippocampal formation in the pilocarpine model of temporal lobe epilepsy—an X-ray fluorescence microscopy study

**DOI:** 10.1007/s00216-012-6425-5

**Published:** 2012-10-04

**Authors:** J. Chwiej, J. Kutorasinska, K. Janeczko, K. Gzielo-Jurek, L. Uram, K. Appel, R. Simon, Z. Setkowicz

**Affiliations:** 1 Faculty of Physics and Applied Computer Science, AGH University of Science and Technology, Al. Mickiewicza 30, 30-059 Krakow, Poland; 2 Institute of Zoology, Jagiellonian University, Ul. Gronostajowa 9, 30-387 Krakow, Poland; 3 Deutsches Elektronen-Synchrotron (DESY), Notkestraße 85, 22607 Hamburg, Germany; 4 Research Centre Karlsruhe, Institut fur Synchrotronstrahlung, Hermann-von-Helmholtz-Platz 1, 76344 Eggenstein-Leopoldshafen, Germany

**Keywords:** Pilocarpine model of epilepsy, Topographic and quantitative elemental analysis, X-ray fluorescence microscopy, Synchrotron radiation

## Abstract

In the present paper, X-ray fluorescence microscopy was applied to follow the processes occurring in rat hippocampal formation during the post-seizure period. In the study, one of the *status epilepticus* animal models of epilepsy was used, namely the model of temporal lobe epilepsy with pilocarpine-induced seizures. In order to analyze the dynamics of seizure-induced elemental changes, the samples taken from seizure-experiencing animals 3 h and 1, 4, and 7 days after proconvulsive agent administration were analyzed. The obtained results confirmed the utility of X-ray fluorescence microscopy in the research of mechanisms involved in the pathogenesis and progress of epilepsy. The topographic and quantitative elemental analysis of hippocampal formations from different periods of epileptogenesis showed that excitotoxicity, mossy fibers sprouting, and iron-induced oxidative stress may be the processes responsible for seizure-induced neurodegenerative changes and spontaneous recurrent seizures occurring in the chronic phase of the pilocarpine model. The analysis of correlations between the recorded elemental anomalies and quantitative parameters describing animal behavior in the acute period of pilocarpine-induced *status epilepticus* showed that the areal densities of selected elements measured in the latent period strongly depend on the progress of the acute phase. Especially important seem to be the observations done for Ca and Zn levels which suggest that the intensity of the pathological processes such as excitotoxicity and mossy fibers sprouting depend on the total time of seizure activity. These results as well as dependencies found between the levels of S, K, and Cu and the intensity of maximal seizures clearly confirm how important it is to control the duration and intensity of seizures in clinical practice.

## Introduction

Intensive development of medicine and biomedical sciences implicates the necessity to search for new investigational tools that would provide more detailed information about biochemical composition of analyzed samples. The unique features of synchrotron radiation, such as high intensity, collimation, and wide spectral range, enable the examination of most subtle biomolecular changes occurring at ranges even less than micrometer [1–6]. Therefore, synchrotron radiation-based techniques such as X-ray crystallography, X-ray fluorescence microscopy, X-ray absorption fine structure spectroscopy, and Fourier transform infrared microspectroscopy play an increasing role in biomedical research [7–14].

Synchrotron radiation-induced X-ray fluorescence microscopy is a very sensitive tool for topographic and quantitative multielemental analysis. It offers spatial resolution and detection limits similar to other methods of elemental analysis such as: microparticle-induced X-ray emission, secondary ion mass spectrometry, or electron probe microanalysis [15–17]. However, comparing to them, it usually does not require any special sample preparation and measurement environment [15].

Trace elements are involved in many processes that may finally lead to atrophy and death of nerve cells in case of different central nervous system (CNS) pathologies [18–21]. Therefore, analysis of their tissue levels and distributions may shed some new light on the mechanisms of CNS diseases of unknown or not fully understood etiology. One of them is epilepsy being the third most common neurological disorder after cerebrovascular and Alzheimer's disease. Epilepsy can either be idiopathic or acquired which means that it is initiated by neurological insults including *status epilepticus*, stroke, or traumatic brain injury. Acquired epilepsy develops in the following three phases: (1) injury, (2) epileptogenesis, and (3) the chronic phase with spontaneous recurrent seizures [22]. Because human tissues for the research of epilepsy can be obtained only postmortem or during surgical resection of *epileptic foci*, the investigations of the most interesting period of epileptogenesis are carried out based on animal models of the disease. Animal models of epilepsy help to understand better the mechanisms leading to spontaneous seizure activity, allow observations of the progress and character of seizures, as well as evaluation of the action of new antiepileptic drugs [23, 24].

Temporal lobe epilepsy (TLE) is the most frequent and medically refractory epilepsy in humans [25–27]. One of the most frequently used and highly isomorphic with human cases of the TLE animal model is one with seizures induced with pilocarpine [28]. Administration of pilocarpine in rats evokes sequential behavioral and electrographic changes that can be divided into three distinct periods: (1) an acute period that builds up progressively into a limbic *status epilepticus*, (2) a silent (latent) period with progressive normalization of EEG and behavior, and (3) a chronic period with spontaneous recurrent seizures [29].

In the present paper, we tried to show how X-ray fluorescence microscopy might support the investigation of the mechanisms leading to seizure-induced neurodegenerative changes of hippocampal formation and to spontaneous seizure activity observed in the chronic period of the pilocarpine model. The main purpose of the study was the analysis of the dynamics of the elemental changes observed in rat hippocampus after administration of proconvulsive agent. In order to accomplish it, the topographic and quantitative elemental analysis was carried out on tissues, taken from animals 3 h (SE3H group) and 1 (SE24H), 4 (SE4D), and 7 (SE7D) days after the pilocarpine injection. Moreover, a correlative analysis was done between recorded elemental anomalies and quantitative parameters describing animal behavior in the acute period of pilocarpine-induced *status epilepticus*, such as intensity of maximal seizures, latency time after pilocarpine injection, and total time of seizure activity within the observation period.

## Materials and methods

All animal experimental procedures were accepted by the Bioethical Commission of Jagiellonian University and were in agreement with the international standards. Adult Wistar rats were obtained from an animal colony of the Department of Neuroanatomy, Institute of Zoology, Jagiellonian University, Krakow. During their whole life, the animals were maintained under conditions of controlled temperature (20 ± 2 °C) and illumination (12:12-h light/dark cycle). A solid diet (Labofeed) and water were available ad libitum.

Seizures were induced in 60-day-old male Wistar rats by i.p. injection of pilocarpine (300 mg/kg; Sigma P6503). Scopolamine methyl bromide (1 mg/kg; Sigma S8502) was injected i.p. 30 min before pilocarpine to reduce its peripheral effects.

The animals were deeply anesthetized using Vetbutal (Polfa) and perfused with 0.9 % saline of high analytical grade 3 h and 1, 4, and 7 days after pilocarpine administration. The brains removed from the skull were first deeply frozen in liquid nitrogen and then cut horizontally in a cryomicrotome into 15-μm-thick sections. The specimens of the dorsal part of the hippocampus [30] were mounted on Ultralene® foil and freeze dried. The numbers of animals representing the analyzed animal groups are specified in Table 1.

**Table 1:** Experimental groups

Group	Pilocarpine injection	Sampling time^a^	Quantity
N		3 h	5
SE3H	+	3 h	4
SE24H	+	24 h	5
SE4D	+	4 days	5
SE7D	+	7 days	16

*N* naive rats

^a^Time of perfusion with physiological saline solution calculated from pilocarpine injection

## Experimental method and apparatus

X-ray fluorescence microscopy was used for the qualitative, quantitative, and topographic elemental analysis. The measurements were carried out at HASYLAB beamline L and the FLUO beamline at ANKA. In both cases, excitation energy of 17 keV was selected and the X-ray beam was focused with polycapillary optics. The diameter of the beam impinging on the sample was 15 and 12 μm, respectively. Silicon drift detectors were used to detect the fluorescence radiation from the sample. The detectors were positioned at the angle of 45° in respect to sample and 90° in respect to the exciting beam. The samples were mapped in two dimensions and the time of single fluorescence spectrum acquisition was 10 s at beamline L and 8 s at the FLUO beamline.

Reference measurements on the following MICROMATTER XRF calibration standards: GaP, KCl, CaF_2_, Fe, ZnTe, Se, and RbI were performed for spectrometer calibration and elemental sensitivities calculations.

## Results

**Fig. 1:**
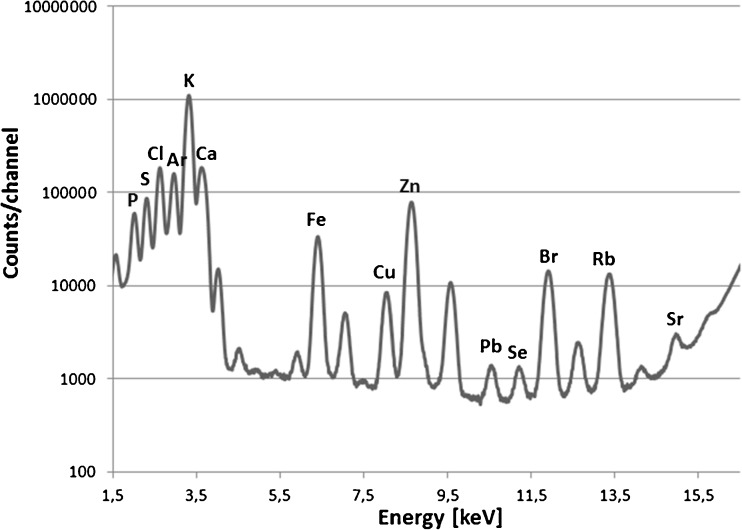
Cumulative spectrum recorded for the hippocampal formation from rat representing SE7D group

The applied measurement conditions allowed to detect elements with atomic numbers between 15 and 38 in the nervous tissue as demonstrated in Fig. 1 which shows the cumulative spectrum of the hippocampal formation.

The analysis of single X-ray fluorescence spectra as well as batch processing of large datasets were carried out using PyMCA software freely available for noncommercial use. The obtained net peak areas of K-α lines of the analyzed elements and the elemental sensitivities evaluated based on the measurements of calibration standards allowed to calculate elemental areal densities for the examined tissue points. Details of the quantitative analysis were presented elsewhere [31]. The comparisons between the examined animal groups were done only for these elements that for all the used experimental conditions, analyzed samples, and measured areas presented the areal densities higher than the detection limits calculated for the tissue samples. Additionally, from further analysis we excluded Cl which originated mainly from physiological saline solution used for perfusion of animals and Pb which was mainly of apparatus origin. Finally, the contents of S, K, Ca, Fe, Cu, and Zn were analyzed in frame of the study.

**Fig. 2:**
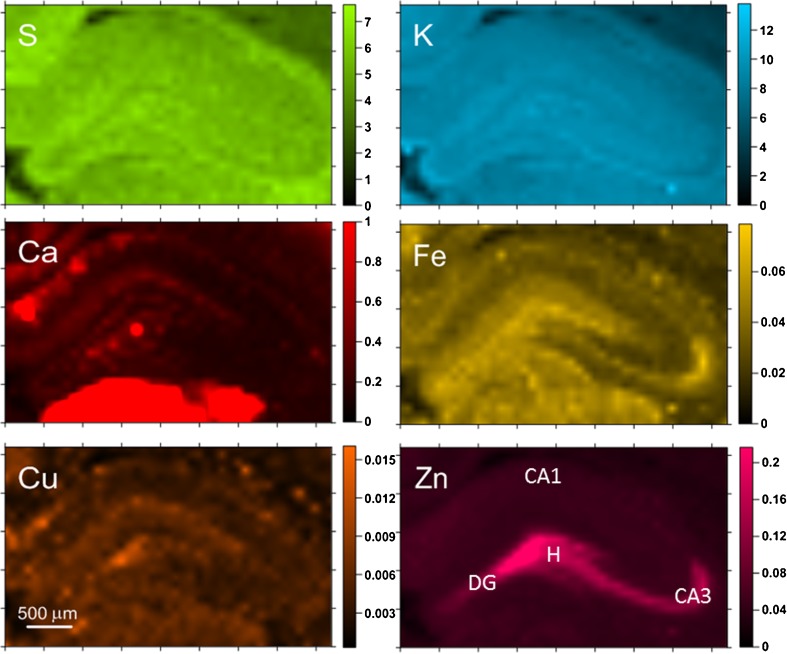
Elemental maps obtained for hippocampal tissue from a selected SE7D epileptic animal. Scales display masses per unit area of the elements in micrograms per square centimeter

**Fig. 3:**
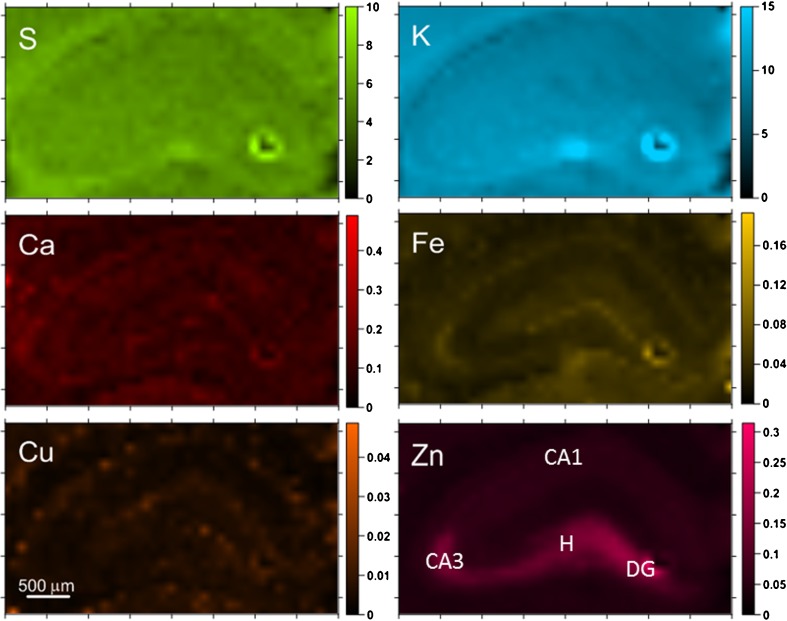
Elemental maps obtained for hippocampal tissue from a selected control animal. Scales display masses per unit area of the elements in micrograms per square centimeter

The next step of the work was the topographic analysis of the examined samples. In Figs. 2 and 3, the results of such analysis done for selected SE7D and control samples are presented.

**Fig. 4:**
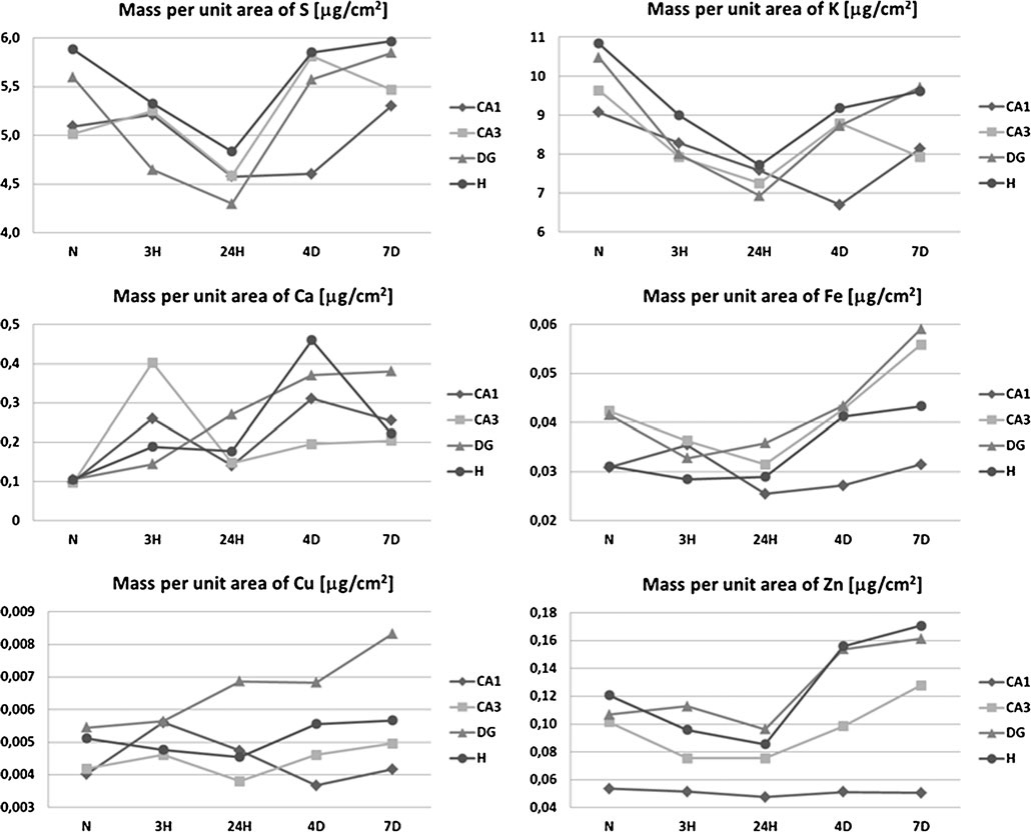
Changes in median values of elemental areal densities for the analyzed hippocampal areas (CA1, CA3, DG, and H) in relation to the time after pilocarpine injection

The comparison of elemental maps with the microscopic views of the scanned tissues allowed identifying the hippocampal areas for further comparison between analyzed animal groups. These were sectors 1 and 3 of the Ammon’s horn (CA1 and CA3, respectively), the dentate gyrus (DG) and hilus of DG (H). For all examined samples, the mean elemental areal densities in the abovementioned areas were calculated. In order to analyze the statistical significance of the differences between animals from different periods of epileptogenesis, the medians of mean values were evaluated for SE3H, SE24H, SE4D, and SE7D groups, as well as naive rats and the obtained results are presented in Fig. 4.

For statistical analysis of the obtained results, nonparametric tests were applied. Such a choice was dictated primarily by the sizes of the experimental groups, which were too little to verify the normality of data distributions. The Mann–Whitney *U* test was applied for statistical evaluation of the differences between animal populations under analysis. In turn, statistically significant dependencies between elemental anomalies and parameters describing animal behavior in the acute phase of pilocarpine-induced *status epilepticus* were determined based on the values of Spearman’s rank correlation coefficients.

**Fig. 5:**
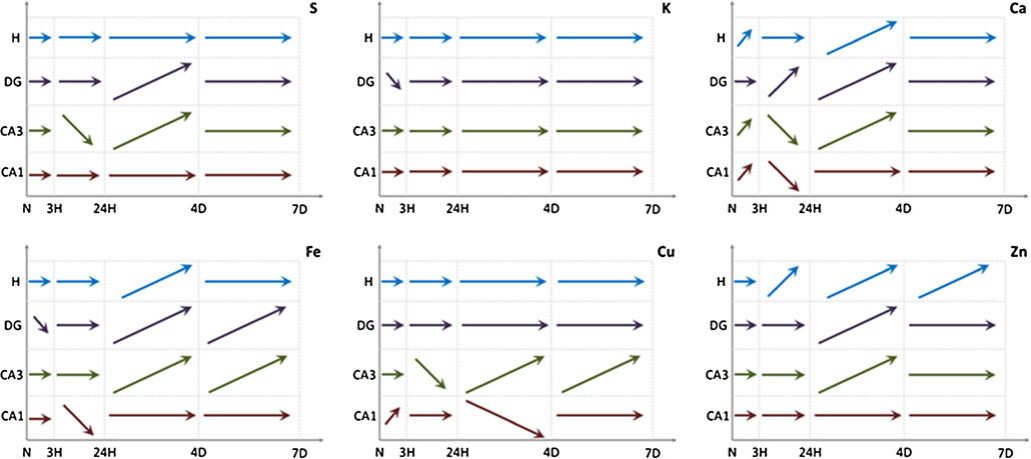
Statistically significant changes in elemental accumulation between subsequent moments after pilocarpine injection

Evaluation of the elements presenting statistically significant differences in accumulation between analyzed groups was done based on *p* values of the *U* test at the significance level of 0.05 and the obtained results are shown in Table 2 and schematically pictured in Fig. 5.

**Table 2:** Statistically significant differences in elemental composition between analyzed animal groups

Elements	SE3H vs. N				SE24H vs. N				SE4D vs. N				SE7D vs. N				SE24H vs. SE3H				SE4D vs. SE24H				SE7D vs. SE4D			
	CA1	CA3	DG	H	CA1	CA3	DG	H	CA1	CA3	DG	H	CA1	CA3	DG	H	CA1	CA3	DG	H	CA1	CA3	DG	H	CA1	CA3	DG	H
S							↓↓**	↓										↓				↑	↑↑					
K			↓*		↓	↓	↓↓	↓↓	↓		↓	↓	↓															
Ca	↑	↑		↑	↑↑	↑	↑↑	↑↑	↑↑	↑↑		↑↑	↑↑	↑↑	↑↑	↑↑	↓	↓	↑			↑↑	↑	↑				
Fe			↓				↓							↑	↑↑	↑↑	↓					↑	↑	↑		↑	↑	
Cu	↑				↑						↑↑				↑			↓			↓	↑				↑		
Zn								↓			↑			↑	↑	↑↑				↑		↑	↑↑	↑↑				↑

*↓ or ↑ decrease or increase in mass per unit area of element at 0.01 <*p-value*<0.05

**↓↓ or ↑↑ decrease or increase in mass per unit area of element at *p-value*<0.01

As one can see in Fig. 5, the level of S does not change in any of the examined areas during the first 3 h after pilocarpine administration. During the next observation period, the areal density of this element decreases in the CA3 sector while between the first and fourth day of the observation, it increases in the CA3 and DG hippocampal areas. In the next examined time period, the level of S does not change, and on the seventh day from the injection of pilocarpine, the areal density of S reaches the value of the control group. Thus, fluctuations of the S content in the analyzed hippocampal areas seem to be temporary effect of pilocarpine-induced seizures.

In all hippocampal areas, despite DG, the content of Ca increases significantly already 3 h after pilocarpine administration. In DG, this rise appears with delay, between the third and twenty-fourth hour, when in the other examined areas the level of Ca decreases (CA1 and CA3) or remains constant (H). In the next time period, the areal density of Ca increases again in CA3, DG, and H hippocampal areas and afterwards remains at a constant level which for all of the analyzed areas is at least twice higher than that recorded for the control group.

Iron areal density decreases during the first 3 h from pilocarpine administration in DG and during the next 21 h of the acute phase in CA1 hippocampal area. Afterwards, during the whole latent period (between the twenty-fourth hour and seventh day from pilocarpine injection), the level of Fe increases in CA3, DG, and H, while for hilus of DG these changes are not statistically significant. Seven days after pilocarpine administration, the content of Fe for these three areas remains higher than the level recorded in control samples.

Accumulation of Zn does not change during the first 3 h from pilocarpine injection in any of the examined hippocampal areas. During the next 21 h of the acute phase, the Zn level starts to increase in H and this tendency persists throughout the remaining 6 days of the observation period. During the first 3 days of the latent phase, the increase in Zn level includes also DG and CA3 hippocampal areas. Seven days after pilocarpine administration, the areal density of Zn in all the three mentioned areas exceeds the level of this element recorded for the control group.

Because of the high mortality rate in rats after pilocarpine injection, only one of examined animal groups was abundant enough to analyze the correlations between the elemental anomalies and quantitative parameters describing animal behavior in the acute phase of pilocarpine-induced *status epilepticus*. The correlative analysis was carried out for the most abundant SE7D group. The behavioral parameters recorded for epileptic animals included: latency time, meaning the time between pilocarpine administration and the first motor seizure sign; intensity of maximal seizures; and total time of seizure activity within the 6-h-long observation period .

The motor seizures intensity was rated on a 6-point scale with respect to the symptoms and their intensity. The scale was used in our previous study [32] and corresponds to that introduced by Racine [33] and widely used in studies on animal models of epilepsy.

1. Light seizures (rated as 0.5 or 1.0)
  For 0.5—immobility, piloerection, salivation, eyes narrowing, face and vibrissae twitching, and ears rubbing with forepaws
  For 1.0—head nodding and chewing movements
2. Intermediate seizures (rated as 1.5 or 2.0)
  For 1.5—clonic movements of forelimbs and mild whole body convulsions, exophthalmia, and aggressive behavior
  For 2.0—rearing and running with stronger tonic–clonic motions including hind limbs, the tail, hypertension, and lockjaw
3. Heavy seizures (rated as 2.5 or 3.0)
  For 2.5—rearing and falling and eye congestion
  For 3.0—loss of postural tone with general body rigidity

The behavioral parameters recorded for the SE7D group are shown in Table 3. In turn, the results of the analysis of the dependencies between them and elemental anomalies measured using X-ray fluorescence microscopy are presented, as Spearman’s rank correlation coefficients, in Table 4. The values statistically significant at the 95 % confidence level are marked.

**Table 3:** The results of behavioral observations recorded for SE7D group

Sample code	TL (min)	MAX	T (min)
NS070	360	0	0
NS071	30	3	220
NS072	300	0.5	80
NS074	360	0	0
NS075	20	2.5	340
NS076	60	1	310
NS079	30	1.5	340
NS0710	30	2.5	310
NS0711	30	1.5	320
NS0712	80	0.5	10
NS0713	360	0	0
NS0714	20	2.5	330
NS0716	210	3	10
NS0717	60	0.5	20
NS0718	360	0	0
NS0719	30	1.5	340

*TL* latency time, meaning the time between pilocarpine administration and the first motor seizure sign, *MAX* intensity of maximal seizures, *T* total time of seizure activity within the 6-h-long observation period

**Table 4:** The Spearman’s rank correlation coefficients obtained at the confidence level of 95 %

Area	Parameter	S	K	Ca	Fe	Cu	Zn
CA1	TL	**0.54**	**0.71**	**−0.50**	0.27	**0.51**	0.43
	MAX	**−0.67**	**−0.64**	0.29	−0.47	**−0.50**	−0.31
	T	−0.30	**−0.63**	**0.73**	−0.18	−0.38	−0.46
CA3	TL	0.30	**0.64**	**−0.55**	−0.13	0.26	−0.06
	MAX	−0.38	**−0.51**	0.35	−0.16	−0.37	−0.19
	T	−0.15	**−0.62**	**0.71**	0.31	−0.26	0.32
DG	TL	−0.10	**0.53**	**−0.53**	−0.38	−0.11	−0.30
	MAX	0.06	−0.26	0.30	0.29	0.14	0.19
	T	0.30	−0.49	**0.74**	0.49	0.15	**0.50**
H	TL	−0.21	**0.62**	**−0.58**	−0.24	0.08	−0.23
	MAX	0.17	−0.37	0.45	0.22	0.10	0.22
	T	0.37	**−0.70**	**0.62**	0.12	−0.11	0.22

*TL* latency time, meaning the time between pilocarpine administration and the first motor seizure sign, *MAX* intensity of maximal seizures, *T* total time of seizure activity within the 6-h-long observation period

Statistically significant correlations coefficients are in bold

Spearman’s rank correlation is a nonparametric alternative to correlation and is typically used when the data do not meet the assumptions about normality, homoscedasticity, and linearity. It is a measure of statistical dependence between two variables which allows easily identifying the strength of correlation within a dataset of two variables. Because in our study we have not got enough data to test the normality and homoscedasticity assumptions of regression and correlation, we decided to use Spearman’s rank correlation to test whether the selected elemental and behavioral parameters covary.

Based on the data from Table 4, one can conclude that 7 days after pilocarpine administration, the levels of selected elements still strongly depend on the progress of the acute phase of pilocarpine-induced *status epilepticus*. Such observations were done mainly for Ca and K. Areal density of Ca in all analyzed areas was negatively correlated with the latency time and positively correlated with the total time of seizure activity within the observation period. For K and all examined hippocampal areas, the exactly opposite relations were observed. Additionally, for this element, negative correlation was noticed between CA1 and CA3 accumulation and intensity of maximal seizures.

The levels of S and Cu were the higher the later the seizure activity occurred after pilocarpine injection and negatively correlated with the intensity of maximal seizures. In turn, Zn areal density, similarly as it was found for Ca, showed (in DG area) positive correlation with the total time of seizure activity.

## Discussion

In the present paper, X-ray fluorescence microscopy was applied to follow the processes occurring in rat hippocampal formation during the period of epileptogenesis. In the study, one of the *status epilepticus* animal models of epilepsy was used, namely the model of TLE with pilocarpine-induced seizures. In order to analyze the dynamics of seizure-induced elemental changes, samples taken from seizure-experiencing animals 3 h and 1, 4, and 7 days after proconvulsive agent administration were examined.

The detailed quantitative elemental analysis of CA1, CA3, DG, and H hippocampal areas and subsequent statistical evaluation showed that excitotoxicity, mossy fibers sprouting, and iron-induced oxidative stress are the processes which may be responsible for seizure-induced neurodegenerative changes and spontaneous seizure activity occurring in the chronic phase of the pilocarpine model. The detailed discussion confirming the thesis is presented below.

A significant decrease in the S content in CA3 hippocampal area detected in the present study 24 h following seizure induction might reflect effects of mitochondrial oxidative stress on cellular concentration of sulfur-containing amino acids and their metabolism [34, 35]. A spectacular consequence of seizures is glutathione depletion (52.9 % of the normal level) in the hippocampus as early as 1 h after limbic *status epilepticus* [36] considerably reducing endogenous antioxidant stores. A subsequent return of the S content to the normal level could be attributed to the onset of reactive processes leading to partial at least, functional recovery of the hippocampal tissue [37]. In this respect, long-term consequences of mitochondrial oxidative stress and their role in epileptogenesis need further exploration [38].

In all hippocampal areas, despite DG, the Ca content increases significantly already 3 h after pilocarpine administration. In DG, this rise appears with a delay between the third and twenty-fourth hours, when in the other examined areas the level of Ca decreases (CA1 and CA3) or remains constant (H). In the next time period, the areal density of Ca increases again in CA3, DG, and H hippocampal areas and remains at a constant level afterwards which is at least a factor of 2 higher for all analyzed areas than for the control group.

An increased Ca level in the acute phase of the pilocarpine model is a result of seizure-induced excessive glutamate release which activates postsynaptic NMDA receptors and triggers receptor-mediated Ca influx [38]. As it was shown in the “Results,” this rise in CA1, CA3 and H hippocampal areas was observed for an earlier period than in DG. Such an observation confirms that DG granular cells present relatively high, comparing to CA1 and CA3 pyramidal cells as well as hilar neurons, resistance to seizure-induced excitotoxicity [25, 39].

Excessive accumulation of Ca in the acute period leads, in turn, to increased glutamate release, which acts on the AMPA/kainate receptors and thus allows further influx of Na^+^ and Ca^2+^ ions into neurons. As a consequence of this process, Mg^2+^ ions, which in the normal conditions block NMDA receptors, are removed. This allows further activation of these receptors through glutamate and Ca influx into postsynaptic cells [40] what we might observe as a secondary increase of the Ca level in CA3, DG, and H hippocampal areas in the latent phase (4 days after pilocarpine administration).

An increase in Ca^2+^ signal was previously recorded in epileptic brain slices and localized in astrocytes [41, 42]. It is necessary to mention that seizures themselves could evoke proliferation of the astroglial cells [43, 44] further reinforcing the total elevation in Ca tissue contents lasting even three postseizure days.

As it was shown in the “Results,” the Fe areal density decreased in the acute and increased in the latent phase after pilocarpine administration. Seven days after SE injection, its levels in CA3, DG, and H are higher than those measured for the control group. The decrease of the Fe areal density during the acute period of pilocarpine-induced SE is probably an effect of seizures-related reversible blood–brain barrier injury [45–48]. In turn, the increased level of Fe observed for the latent phase indicates that this element may play an important role in the processes leading to seizure-induced neurodegenerative changes of the hippocampal formation and pathogenesis of spontaneous seizures in the chronic phase of pilocarpine model. Iron, promoting the production of free radicals, potentiates the effect of oxidative stress. As a transitive metal it catalyzes Fenton’s reaction leading to the creation of highly reactive hydroxyl radicals. Clinical studies show that brain injuries, occurring with extravasation of red blood cells, hemolysis, and the deposition of iron compounds, often result with the appearance of posttraumatic seizures [49]. Similar effects are obtained experimentally by injection of iron salts or hem in particular brain areas [50, 51]. In both cases, the appearance of epileptic seizures is connected with increased, through accumulating iron, production of free radicals and occurring as a result of their action lipid peroxidation [50, 52, 53].

Seven days after pilocarpine administration, a significant increase of Zn accumulation was observed in CA3, DG, and H hippocampal areas but not in CA1 region. An increased areal density of Zn in the latent phase is probably a consequence of mossy fibers sprouting which means synaptic reorganization occurring in the inner molecular layer of DG. In the normal conditions, mossy fibers, being the axons of DG granule cells, target to pyramidal cells of CA3 region. However, after epileptic injury they sprout into the inner molecular layer and hilus, as well as stratum oriens in CA3 [54] and may give significant input in the hyperexcitability and epileptic seizures occurring both in humans and in animal models of the disease [55–59]. Because large terminals of mossy fibers of dentate granule cells contain the highest amounts of Zn in the brain [60], the new collaterals sprouted in the molecular layer are probably responsible for the higher content of this element, which is observed in epileptic animals from the latent phase.

Ongoing seizures may also trigger Zn^2+^ release both from synaptic terminals [61–63] and from intracellular stores [64, 65]. However, for none of the analyzed hippocampal areas, we observed an excessive accumulation of Zn in the acute phase of pilocarpine-induced *status epilepticus* (SE3H and SE24H groups). Twenty-four hours after pilocarpine administration, the level of Zn in the hillus of DG was even lower than the level found for naive control animals. Therefore, the phenomena of mossy fibers sprouting seems to be much better explanation for an increase in Zn areal density occurring in DG, H, and CA3 hippocampal areas 7 days after seizure induction.

Besides the dynamics of seizure-induced elemental changes in the hippocampal formation, the correlations between elemental anomalies and quantitative parameters describing animal seizure behavior in the acute period were examined. The statistical analysis based on Spearman’s rank correlation coefficients was carried out for the most abundant SE7D group. The obtained results showed at the significance level of 0.05 that the areal densities of selected elements measured in the latent period strongly depend on the progress of the acute phase of pilocarpine-induced *status epilepticus*. Especially important seem to be the recorded changes in Ca and Zn levels which suggest that the intensity of the processes such as excitotoxicity and mossy fibers sprouting depend on the total time of seizure activity. These results as well as further relations found between the levels of S, K, and Cu and the intensity of seizures clearly confirm how important it is to control the duration and intensity of seizures in clinical practice.

## Conclusions

The results presented in this paper confirmed the utility of X-ray fluorescence microscopy in investigations of mechanisms involved in the pathogenesis and progress of epilepsy. The topographic and quantitative elemental analysis of the hippocampal formations at different stages of epileptogenesis showed that excitotoxicity, mossy fibers sprouting, and iron-induced oxidative stress may be the processes underlying seizure-induced neurodegenerative changes and spontaneous seizure activity occurring in the chronic phase of pilocarpine model.
